# University students and HIV in Namibia: an HIV prevalence survey and a knowledge and attitude survey

**DOI:** 10.1186/1758-2652-15-9

**Published:** 2012-02-22

**Authors:** Ingrid H de Beer, Huub C Gelderblom, Onno Schellekens, Esegiel Gaeb, Gert van Rooy, Alta McNally, Ferdinand W Wit, Rinke de Wit F Tobias

**Affiliations:** 1 PharmAccess Foundation Namibia, Windhoek, Namibia; 2 Master’s Program in Global Public Health, New York University, New York, NY, USA; 3 Current address: Hasso Plattner Research Laboratory, University of KwaZulu Natal, Durban, South Africa; 4 Current address: International Trachoma Initiative, Task Force for Global Health, Emory University, Atlanta, USA; 5 PharmAccess Foundation, Amsterdam, The Netherlands; 6 Namibia Institute of Pathology, Windhoek, Namibia; 7 Multidisciplinary Research Center, University of Namibia, Windhoek, Namibia; 8 Polytechnic of Namibia, Windhoek, Namibia; 9 Center for Poverty-related Communicable Diseases, Academic Medical Center of the University of Amsterdam, Amsterdam, The Netherlands; 10 Center for Internal Medicine, Center for Infection and Immunity (CINIMA), Amsterdam, The Netherlands; 11 Amsterdam Institute for Global Health and Development (AIGHD), Amsterdam, The Netherlands; 12 Academic Medical Center of the University of Amsterdam, Amsterdam, The Netherlands

## Abstract

**Background:**

With an overall adult HIV prevalence of 15.3%, Namibia is facing one of the largest HIV epidemics in Africa. Young people aged 20 to 34 years constitute one of the groups at highest risk of HIV infection in Namibia. However, little is known about the impact of HIV on this group and its access to healthcare. The purpose of this study was to estimate HIV prevalence, to assess the knowledge of and attitudes towards HIV/AIDS, and to assess access to healthcare among university students in Namibia.

**Methods:**

We assessed HIV/AIDS knowledge and attitudes, HIV prevalence and access to healthcare among students at the Polytechnic of Namibia and the University of Namibia. HIV prevalence was tested through anonymous oral fluid-based tests.

**Results:**

Half (n = 2790/5568) of the university students and 45% (n = 2807/6302) of the Polytechnic students participated in the knowledge and attitudes surveys. HIV/AIDS knowledge was reasonable, except for misperceptions about transmission. Awareness of one's own HIV status and risks was low. In all, 55% (n = 3055/5568) of university students and 58% (n = 3680/6302) of Polytechnic students participated in the HIV prevalence survey; 54 (1.8%) university students and 103 (2.8%) Polytechnic students tested HIV positive. Campus clinics were not the major providers of healthcare to the students.

**Conclusions:**

Meaningful strategies addressing the gap between knowledge, attitude and young people's perception of risk of HIV acquisition should be implemented. HIV prevalence among Namibian university students appears relatively low. Voluntary counselling and testing should be stimulated. Efforts should be made to increase access to healthcare through the campus clinics.

## Background

Namibia in southern Africa has approximately 2.2 million inhabitants and is classified as a middle-income country. The Namibian health system has both a public health service through the Ministry of Health and Social Services (MoHSS) and a relatively well-established private health sector. However, as the country is experiencing a large HIV epidemic, HIV/AIDS places a significant burden on the Namibian health system [1].

The overall adult HIV prevalence in Namibia is estimated at 15.3%, which is among the highest in the world [1]. The HIV prevalence among pregnant women is 19.9% [2]. The estimated HIV prevalence is 10.3% among 15- to 24-year-old females, and 3.4% among 15- to 24-year-old males [1]. In 2008, it was estimated that 204,000 Namibians live with HIV, with an estimated 39 new infections occurring every day, 44% of which are in young people between the ages of 15 and 24 years [3].

AIDS has been the most prominent cause of death in Namibia since 1996, and in 2007, was the cause of 25% of all deaths [4]. In recent years, comprehensive voluntary counselling and testing (VCT) and HIV treatment programmes have been established in Namibia [5]. In 2009, 66 public antiretroviral treatment (ART) sites were operational throughout the country. ART coverage increased from 3% to 60% between 2003 and 2007. Projections for coverage until 2013 stand at 80%. By March 2008, 50,600 people, including approximately 8,000 through the private sector, were receiving ART [3].

According to recent studies [2,5], young people aged 20 to 34 years constitute one of the groups at highest risk of HIV infection in Namibia. This age group forms about 25% of the Namibian population. Overall, the level of education in Namibia is high. According to the last demographic and health survey, in 2006/2007, more than half of the 20- to 34-year-old group attained the secondary level at school and up to 10% reached a higher educational level [6]. University students form an important constituency in interventions against HIV and AIDS. They are also identified as an interesting target group, as they represent the future leaders and economic backbone of the country.

The Polytechnic of Namibia and the University of Namibia, both located in Windhoek, are the two largest tertiary education institutions in the country, educating more than 95% of Namibian university students. These institutions provide primary healthcare and curative services to the students on campus. They give family planning and health education on sexually transmitted infections, such as HIV/AIDS. However, to our knowledge, no data exist on the HIV prevalence at Namibian institutions of higher learning, or on the impact of HIV and access to healthcare of university students. Few studies have focused on students as a group in sub-Saharan Africa [7-13].

The purpose of this study was: (1) to assess students' knowledge of and attitudes towards HIV/AIDS; (2) to estimate HIV prevalence among university students; and (3) to assess their access to healthcare. We report that among university students in Namibia, we found a reasonable overall HIV knowledge, but identified some gaps. We also found relatively low overall HIV prevalence, although it was high in some sub-groups, and low use of existing campus health facilities.

## Methods

### Study population

The target group population consisted of all students of the University of Namibia (UNAM) and the Polytechnic of Namibia in Windhoek, comprising more than 11,800 students.

### Surveys

Two surveys were conducted separately: a survey on knowledge and attitudes (KA) towards HIV/AIDS and an HIV prevalence survey. The surveys were conducted from 6 to 10 August 2007 at UNAM and from 10 to 17 September 2007 at the polytechnic. HIV/AIDS knowledge and attitudes (KA) were evaluated using a self-administered 16-question quantitative survey. This survey also assessed healthcare access.

Namibian nurses contracted by PharmAccess Foundation collected the HIV samples, which were analyzed by laboratory technicians from the Namibia Institute of Pathology. HIV antibody status was assessed using the oral fluid-based OraQuick Rapid HIV-1/2 Antibody Test (OraSure Technologies, Inc., Bethlehem, PA ["OraQuick"]) [14]. The OraQuick is an FDA-approved, non-invasive, rapid diagnostic test that is suitable for epidemiological purposes, and has been officially validated by the Namibian Institute of Pathology [14] and was approved by the MoHSS for surveillance purposes in 2006.

### Organization of the KA and HIV prevalence surveys

Both institutions implemented broad-scale awareness campaigns using campus radio, posters, flyers and information boards prior to the surveys to raise awareness and encourage participation.

Participation in both surveys was voluntary and anonymous. Students received an explanation about the purpose and objectives of the study before being asked for consent and to fill in the questionnaire.

The surveys were conducted at strategically placed sites throughout the Windhoek campuses of UNAM and the Polytechnic, over a period of five days from 07h00 to 20h00, in order to facilitate the participation of as many students as possible, irrespective of location or class schedule.

Test sites were separated into three separate areas to ensure confidentiality: 1) an area where students would fill out the KA survey; 2) an area where the oral fluid swab for the HIV survey was taken by a nurse; and 3) a laboratory. When students participated in both surveys, the results of the KA survey were linked to the results of the HIV prevalence survey using an anonymous barcode system.

### Informed consent and ethical clearance

The surveys were performed at the request of the management committees of the university and the polytechnic and in coordination with respective student representative platforms for operational purposes to inform the institutions' HIV programmes. The study interventions were approved by the participating institutions ethical committees as a part of the HIV management programme. Individual students only participated voluntarily in HIV testing after oral informed consent. HIV testing was performed anonymously and no results were returned to participating individuals. No individual identifiers were collected. Those students who wished to know their HIV status were referred to VCT centres established in Windhoek. The analysis of data for this paper was conducted after the operational surveys of the individual institutions using existing anonymous data.

### Statistical analysis

Graphical representation and statistical analysis were performed using GraphPad Prism version 4.0b for Macintosh (GraphPad Software, San Diego, CA, USA), Microsoft Excel 2004 for Mac (Microsoft, Seattle, WA, USA), and SPSS version 16 for Macintosh (SPSS Inc., Chicago, IL, USA). Statistical analysis was done using the Chi-square test for categorical variables. A two-sided p value < 0.05 was considered statistically significant.

## Results

### Participation in the KA survey

Half (n = 2790/5568) of the UNAM students and 45% (n = 2,807/6,302) of the Polytechnic students participated in the KA surveys (Table 1). The participation rate in the KA survey was significantly higher among female students at both institutions: 65% of female UNAM students compared with 40% of male UNAM students (p < 0.0001, Chi-square), and 51% of female Polytechnic students compared with 39% of male Polytechnic students (p < 0.0001, Chi-square) (Table 2).

**Table 1:** Number of participants in KA survey and/or HIV prevalence survey

Institution		HIV survey yes		HIV survey no		Total	
UNAM	KA yes	**2,789**	(50%)	1	(0%)	2,790	(50%)
	
	KA no	266	(5%)	2,512	(45%)	2,778	(50%)
	
	Total	3,055	(55%)	2,513	(45%)	5,568	(100%)

Polytechnic	KA yes	**2,706**	(43%)	101	(1.6%)	2,807	(45%)

	KA no	974	(15%)	2,521	(40%)	3,495	(55%)

	Total	3,680	(58%)	2,622	(42%)	6,302	(100%)

**Table 2:** Participation of students in the HIV prevalence survey and KA survey according to gender and institution

	Total (n)	Participated in HIV survey		Participated in KA survey	
		
		n	%	n	%
Female students UNAM	3,053	1,950	(67%)	1,792	(65%)

Male students UNAM	2,515	1,105	(44%)	998	(40%)

All students UNAM	5,568	3,055	(55%)	2,790	(50%)

Female students polytechnic	3,042	2,141	(71%)	1,542	(51%)

Male students polytechnic	3,260	1,539	(44%)	1,267	(39%)

All students polytechnic	6,302	3,680	(58%)	2,807	(45%)

Participation rates in the HIV survey and KA survey were significantly higher among female students at both UNAM and the Polytechnic, p < 0.0001, Chi-square

Of the 5,597 students who participated in the KA survey, most were females (60%). The age of the participants ranged from 14 to 53 years (mean = 21.9+/- 4.3); a large majority (93%) were 18 to 30 years old. Among the respondents, 30% were first-year students, 27% were second-year, 20% were third-year, and 23% were fourth-year or more.

### Outcome of the KA survey

Basic knowledge of HIV/AIDS was good: 95% of UNAM and 93% of Polytechnic respondents knew what HIV/AIDS was; and 97% of UNAM and 96% of Polytechnic correctly defined the difference between HIV and AIDS. Knowledge of HIV prevention appeared high as 92% of all respondents knew that using a condom could protect against HIV infection. However, a number of respondents had misperceptions about transmission of HIV by deep kissing (50% UNAM, 44% Polytechnic), witchcraft (26% UNAM, 27% Polytechnic) and shaking hands (14% UNAM, 15% Polytechnic). Almost all respondents knew that HIV could not be transmitted by sharing food with an HIV-positive person (98% UNAM, 97% Polytechnic).

Knowledge of treatment possibilities of HIV/AIDS appeared high: 90% of UNAM and 86% of polytechnic respondents indicated that they knew that there was no treatment that can eradicate HIV/AIDS from a human's body, that traditional healers could not remove HIV from a human's body (96% UNAM, 94% Polytechnic), that a person with tuberculosis was not always HIV positive (95% UNAM, 93% Polytechnic) and that ART could enable HIV-infected people to live longer, healthier lives (94% UNAM, 89% Polytechnic).

Television/radio (73% UNAM, 66% Polytechnic), print media (70% UNAM, 61% Polytechnic) and health institutions (66% UNAM, 64% Polytechnic) were mentioned by most students as major sources of information about HIV/AIDS. Fewer students mentioned information on campus (45% UNAM, 27% Polytechnic) and the campus nurse/counsellor (15% UNAM, 11% Polytechnic) as major sources of information on HIV/AIDS.

The general attitude towards people living with HIV/AIDS was positive for the majority of respondents, indicating that they would be willing to attend classes with an HIV-positive fellow student (95% UNAM, 94% Polytechnic), or attend lectures presented by an HIV-positive lecturer (96% UNAM, 95% Polytechnic). However, only 75% of UNAM and 77% of Polytechnic respondents indicated that they would eat food that was prepared by an HIV-positive person. Although 50% of UNAM and 54% of Polytechnic respondents had indicated that they knew HIV could not be transmitted through deep kissing, only 31% of UNAM and 37% of Polytechnic respondents indicated that they themselves would actually kiss an HIV-positive person.

Awareness of the existence of the institutional HIV/AIDS programmes was moderate (72% UNAM, 71% Polytechnic). Highest on the list of programme services that students desired were availability of counselling (63% UNAM, 59% Polytechnic), HIV/AIDS-related information (51% UNAM, 56% Polytechnic), support for HIV-positive people (64% UNAM, 50% Polytechnic) and condom distribution (36% UNAM, 36% Polytechnic).

### Participation in the HIV prevalence survey

In all, 55% (n = 3055/5568) of UNAM students and 58% (n = 3,680/6,302) of Polytechnic students participated in the HIV prevalence survey (Table 1). The participation rate in the HIV surveillance was significantly higher among female students (67% of female UNAM students compared with 44% of male UNAM students, p < 0.0001, Chi-square, and 71% of female Polytechnic students compared to 47% of male Polytechnic students, p < 0.0001, Chi-square) (Table 2). Participation was especially low (22%) among part-time male Polytechnic students (data not shown). The participation rate was higher in full-time Polytechnic students than in part-time Polytechnic students (p < 0.0001, Chi-square, data not shown).

### Outcome of the HIV prevalence survey

**Figure 1:**
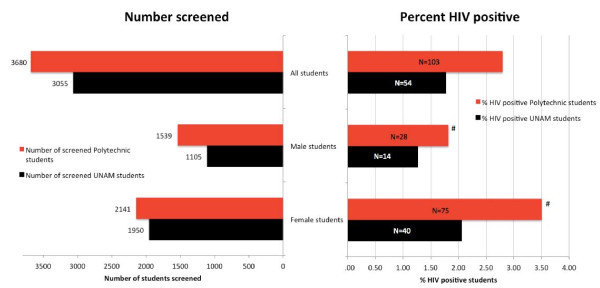
**Numbers of screened students and HIV prevalence, according to gender and institution**. The size of the bars on the right depict the prevalence. The absolute numbers of HIV-positive students are depicted in the bars as "n = ...". ^#^The HIV prevalence was significantly higher among female students, compared with male students, at the polytechnic, p = 0.031, Chi-square.

**Figure 2:**
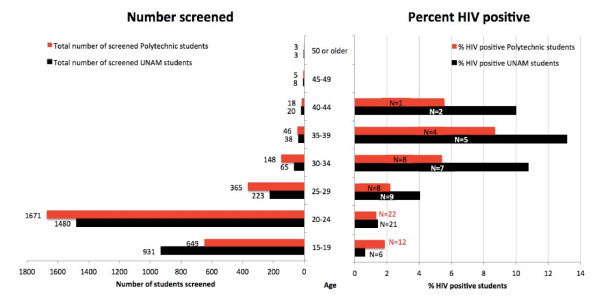
**Number of students screened, and distribution of HIV-positive students according to age and institution**. The size of the bars on the right depict the prevalence. The absolute numbers of HIV-positive students are depicted in the bars as "n=...". In total, 2,905 of 3,680 (79%) participating Polytechnic students and 2,774 of 3,055 (91%) participating UNAM students disclosed their age and were included in this analysis. Note that although the overall percentage of HIV-positive students was higher at the Polytechnic with 2.8%, compared with 1.8% at UNAM, the percentage of HIV-positive students at the Polytechnic who disclosed their age was lower than among the UNAM students who disclosed their age. See also Figure 3.

Of the 3,055 UNAM students and 3,680 Polytechnic students who participated in the anonymous HIV prevalence surveillance, 54 UNAM students (1.8%) and 103 Polytechnic students (2.8%) tested HIV positive (Figure 1). The HIV percentage appeared higher in female UNAM students (2.1%, n = 40/1950) than in male UNAM students (1.3%, n = 14/1105), but this difference was not statistically significant (p = 0.15, Chi-square) (Figure 1). The HIV prevalence was significantly higher in female Polytechnic students (3.5%, n = 75/2141) than in male Polytechnic students (1.8%, n = 28/1539; p = 0.0031, Chi-square) (Figure 1). HIV prevalence was three times as high in part-time Polytechnic students (5.4%, n = 55/1016) than in full-time Polytechnic students (1.8%, n = 47/2653; p < 0.0001, Chi-square, not shown) and highest in part-time female Polytechnic students (6.0%, n = 42/704, data not shown). HIV prevalence increased according to age in both institutions with a peak in the 35-39 age group (Figure 2).

### Behaviour and practices relating to own HIV status

The majority of students are unaware of their HIV status and risks. Overall, 46% of UNAM and 42% of Polytechnic respondents reported knowing their HIV status; 64% (n = 32/50) of the UNAM respondents (significantly higher than HIV-negative students who completed the KA survey, p = 0.0015, Chi-square) and 53% (n = 24/45, p = not significant compared with HIV-negative students who completed the KA survey) of the Polytechnic respondents who tested HIV positive during the HIV prevalence survey (and who also completed the KA survey) reported that they knew their status.

Only 40% of UNAM and 39% of Polytechnic respondents indicated that they thought that they were at risk of becoming infected with HIV. Of the respondents who tested positive for HIV during the prevalence surveillance, 52% of UNAM and 54% of Polytechnic respondents indicated that they believed that they were at risk of contracting HIV, suggesting that HIV-positive students are better aware of their status and may be more aware of present or past risk behaviour.

### Healthcare access

With respect to healthcare delivery, students mentioned state hospitals (38% UNAM, 41% Polytechnic), private doctors (40% UNAM, 38% Polytechnic) and state clinics (26% UNAM, 36% Polytechnic) as their primary access points. The campus clinics were the primary source of healthcare for 39% of UNAM respondents (third-ranked provider), and 18% of Polytechnic respondents (fourth-ranked provider).

## Discussion

In this study among more than 5,000 university students in Namibia, we found: that overall knowledge about HIV/AIDS prevention, transmission and treatment was high, although there were some important misperceptions; that HIV prevalence among respondents was lower than expected (1.8% at UNAM and 2.8% at the Polytechnic); and that campus health facilities were underused.

The level of overall **knowledge about HIV/AIDS **prevention, transmission and treatment is encouraging. Studies from other higher institutions in Africa show similar results, keeping in mind that the respondents had at least secondary level of education and that information campaigns about HIV/AIDS had been conducted in Namibia [8,15]. However, as in other studies performed in sub-Saharan African countries [9,16], we observed some important misperceptions, such as the belief that HIV can be transmitted by deep kissing, witchcraft and shaking hands. Overall attitudes towards people living with HIV/AIDS were positive, except for eating food prepared by an HIV-positive person or kissing an HIV-positive person. As Tebourski *et al *demonstrated [16], there was a discrepancy between the knowledge that HIV cannot be transmitted in certain ways and willingness to engage in that behaviour. These knowledge and attitude issues should be addressed in HIV/AIDS educational campaigns, and meaningful strategies that address the gap between knowledge, risky sexual behaviour and young people's perception of their vulnerability to AIDS must be implemented [8].

An alarming majority of HIV-positive and HIV-negative students appeared to be unaware of their **HIV status **and their risk of acquiring or transmitting HIV. Many students (28% at UNAM and 29% at the Polytechnic) did not know of the institutional HIV/AIDS awareness programmes, and a large majority of students obtained information about HIV/AIDS primarily from media rather than from the university. This suggests that awareness efforts should be improved, that educational efforts at the university should be increased [9], and that VCT should be further encouraged.

**Access to healthcare **was not optimal. The campus clinics were underused, particularly at the Polytechnic (fourth-ranked provider). Several explanations can be stated. First, the campus clinics are now financed as part of the overall running expenses of the institution; no guarantee of quality of medical services can be offered. Second, even if seeking care at overburdened public facilities away from campus is time consuming and expensive, students may do not want to change their habits. Efforts should be made to increase access to healthcare through the campus clinics.

The overall **HIV prevalence **among students was higher at the Polytechnic (2.8%) than at the university (1.8%), but appeared to be lower than expected, based on Namibian population HIV prevalence data (3.7% and 6.5% in persons aged 20-24 years and 25-29 years, respectively) [17]. The difference in overall HIV prevalence at the Polytechnic and UNAM may be related to the different demographics of the two student populations.

Two biases may be present. The first is the tertiary education level of the respondents in this study versus the general population that is assessed in the national surveys [1]. A recent household survey in Windhoek found that the HIV prevalence was lower among those who had finished secondary or higher education [17].

Second, only 57% of all students participated in the HIV prevalence survey. Refusal to participate can generate a bias known as "volunteer bias", which limits the ability to generalize research findings and jeopardizes the validity of research outcomes [18]. In our study, for example, there was 68% participation in full-time female Polytechnic students, of whom 2.3% were HIV positive, versus 22% in part-time male Polytechnic students, of whom 4.8% were HIV positive. Hence, biases may have been introduced that most probably lead to underestimations of actual HIV prevalence.

In a recent household survey in Windhoek, it was estimated that the HIV prevalence amongst non-survey participants was four times higher than among participants [19]. If we assume that the HIV prevalence among the non-participating population of students is four times higher than among those who participated, the HIV prevalence rates would be 4.2% at UNAM and 6.3% at the Polytechnic, which is comparable to the national prevalence data [5]. This also reflected in a sub-analysis of the Polytechnic data where we compare HIV prevalence according to the amount of information that students provided: many Polytechnic students who participated in the HIV prevalence survey did not provide their age (2,905 of the 3,680 students in the HIV survey, 79%, but this sub-set included only 55 of the 103 HIV-positive students, 54%), or participate in the KA survey (2,706 of the 3,680 students in the HIV survey, 74%, also participated in the KA survey, including 45 of the 103 HIV-positive students,44%).

**Figure 3:**
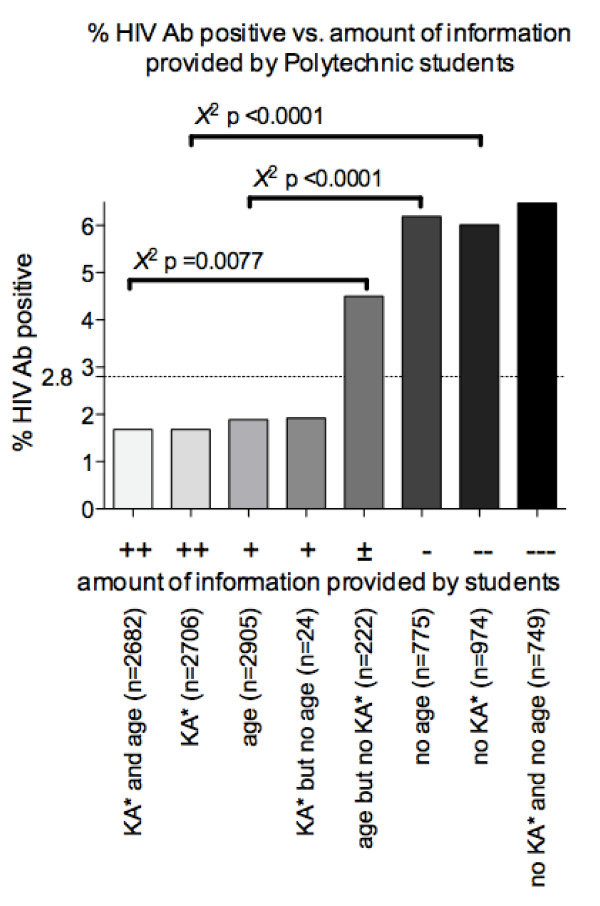
**HIV prevalence in relation to the amount of information provided by 3,680 polytechnic students who participated in the HIV prevalence survey**. The overall HIV prevalence among the 3,680 Polytechnic students was 2.8%.

Most of the 55 HIV-positive students who disclosed their age were in the 20-24-year age group. Given the fact that relatively fewer HIV-positive students (percentage wise) provided their age or participated in the KA survey, it is difficult to determine a relationship between age and HIV status from the present data. This is even more evident when the HIV prevalence is calculated in sub-groups of respondents based on the amount of additional information they provided. Students who participated in the KA survey and also provided their age had a lower HIV prevalence, and students who did not provide any additional information had a significantly higher HIV prevalence (Figure 3, Chi-square).

The KA study may have been biased as it was based on self-reported behaviours. Responses may have been affected by memory bias or social desirability bias. Although social desirability bias tends to be less when using self-administrated questionnaires compared with face-to-face questionnaires [20], it may still result in underestimating sexual risk behaviour and attitudes regarding HIV [21,22].

## Conclusions

In conclusion, at two universities in Namibia, we found that students had moderate to good HIV/AIDS knowledge, yet also had some important misperceptions about HIV/AIDS treatment and transmission, and low knowledge of their own HIV status. HIV prevalence was relatively low, but may be underestimated. There was considerable interest in institutional HIV awareness programmes. The campus clinics were underused. These findings motivate continued and intensified prevention and education initiatives through institutional HIV/AIDS awareness programmes.

## Competing interests

The authors declare that they have no competing interests.

## Authors' contributions

IHdB designed and coordinated the study, participated in the data analysis, and drafted the manuscript. HCG participated in the data analysis, performed the statistical analysis, and drafted the manuscript. OS participated in the design and coordination of the study, and helped draft the manuscript. EG, GvR, AMcN and FWW participated in the design and coordination of the study, and participated in the data analysis. TFRdW conceived of the study, participated in its design and coordination, and helped draft the manuscript. All authors read and approved the final manuscript.
